# Supplementary figures and images for: iCVS—Inferring Cardio-Vascular hidden States from physiological signals available at the bedside
Source: PLoS Comput Biol. 2023 Sep 5;19(9):e1010835. doi: 10.1371/journal.pcbi.1010835 (PMC10503777; doi:10.1371/journal.pcbi.1010835)

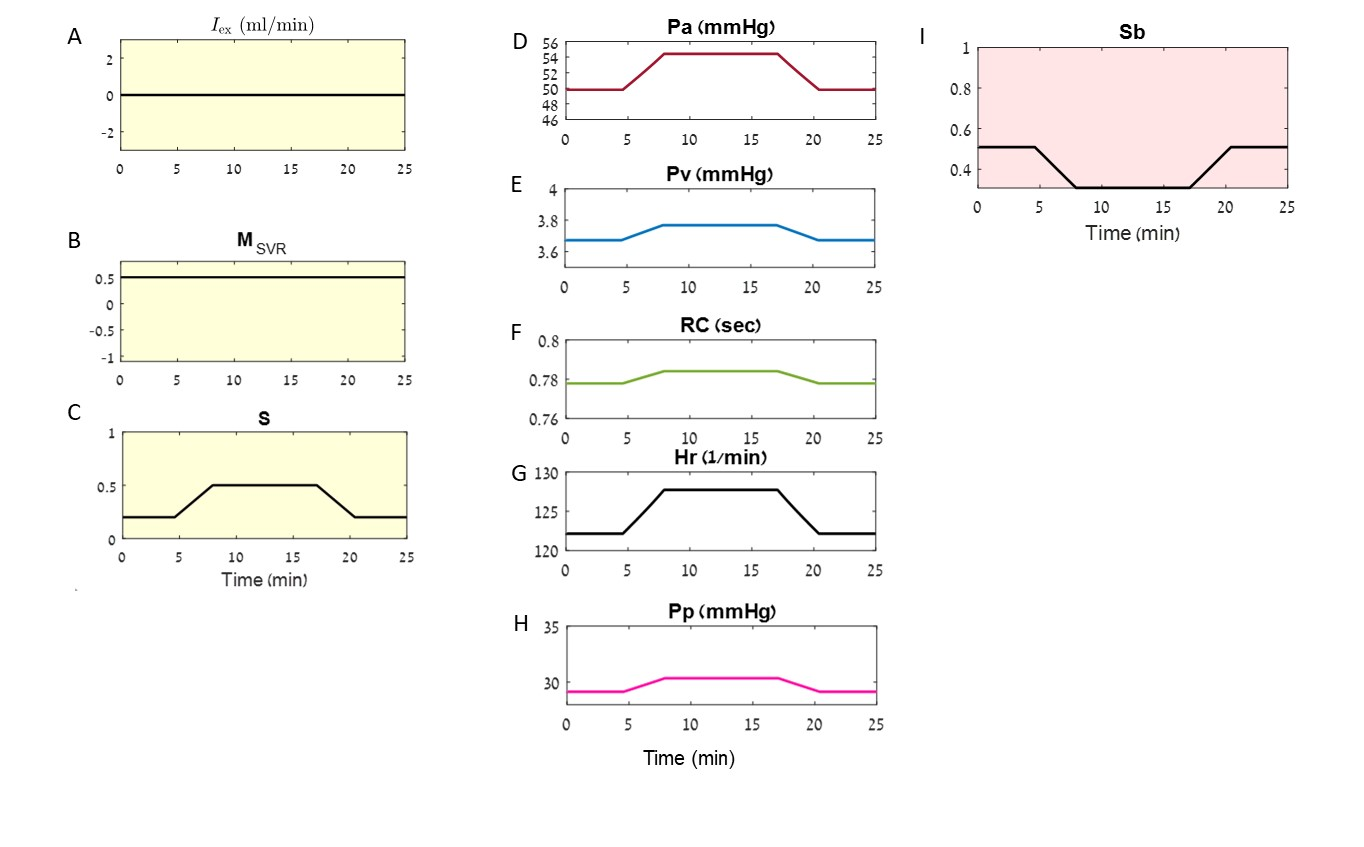

Supplement: S1 Fig — demonstrates the effect of S (independent autonomic control) on the cardio-vascular dynamics in a simulation of the iCVS model. In response to an increase in S, arterial pressure, venous pressure, vascular resistance, and pulse pressure increase as well. The hidden parameters are fixed and the observables are simulated accordingly (see Methods). A. Time dependent intra vascular volume change—zero in this simulation. B. Time course of MSVR—constant in this simulation. C. Time dependent magnitude of the independent autonomic control (S) which does not depend on the cardio-vascular state. D-H The resulting observables: D. Arterial pressure (Pa), E. Venous pressure (Pv), F. Peripheral resistance multiplied by arterial compliance (RC). G. Heart rate (Hr), H. Pulse pressure (Pp). I. Time dependent magnitude of the baro-reflex (Sb) which depends on the arterial blood pressure. (TIFF) [file pcbi.1010835.s001.tiff]

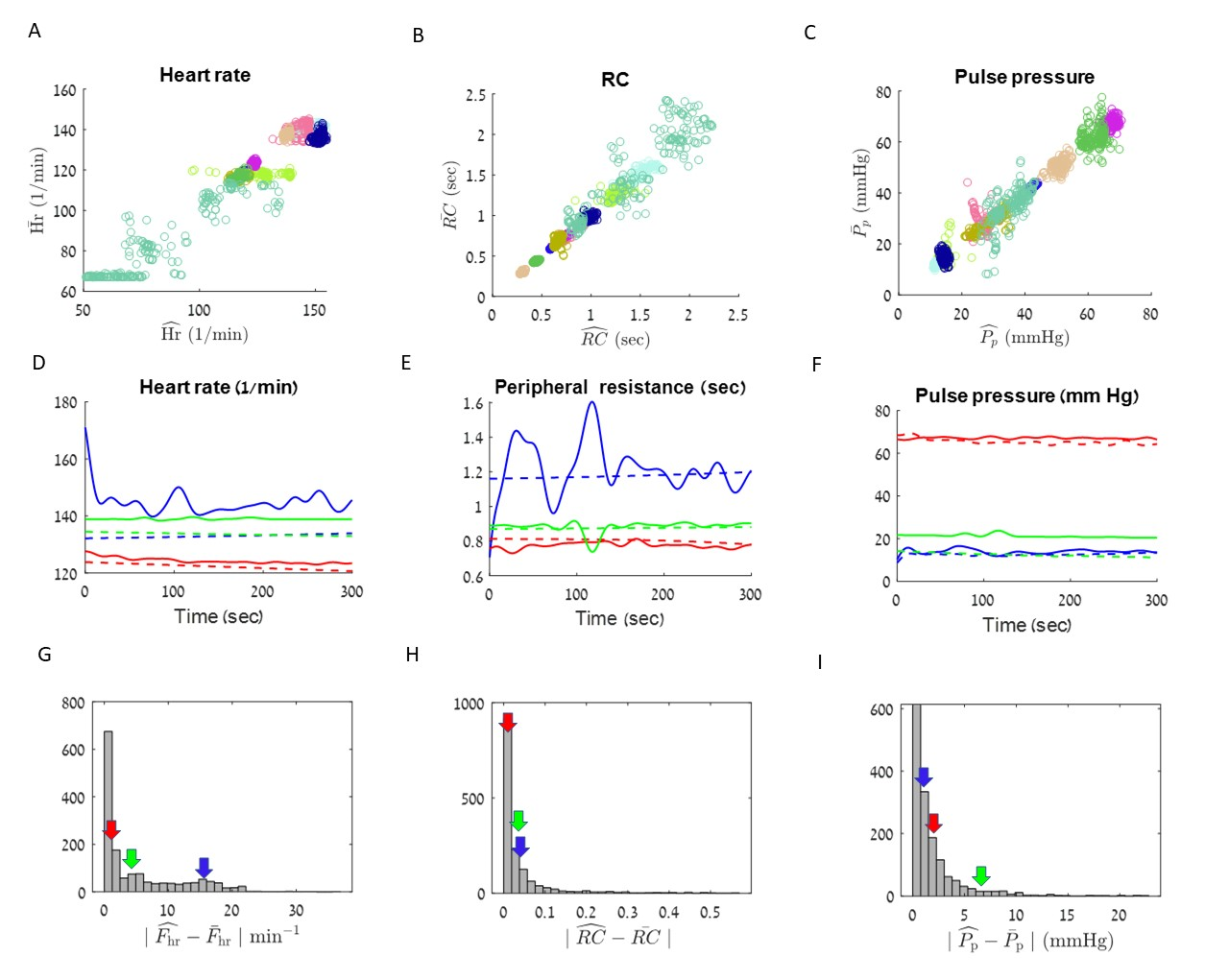

Supplement: S2 Fig — A-C. Comparison between the measured value (horizontal axis) and estimated value (vertical axis). Each point represents the value of a given measurement (A—heart rate, B—peripheral resistance, C—pulse pressure) in a specific time point. The time points which are presented are a randomly chosen subset that constitute 1% of the whole data-set which is used in Fig 9. D—F. Comparison between observed data and reconstructed values during a segment of 500 seconds. Green—patient number 2, blue—patient number 3 and red—patient number 6 (see Table 3). Continuous line—observed data: Hr^ (D), RC^ (E) and Pp^ (F). Dashed line—reconstructed value: Hr¯ (D), RC¯ (E) and Pp¯ (F). G-I. Histograms of the absolute values of the differences between the measured and estimated variables of heart rate (G), peripheral resistance (H) and pulse pressure (I). The dataset is same as presented in panels A-C. The arrows denote the mean differences for each of the patients presented in panels D-F using the same color scheme. Using the estimated parameters which are found by the optimization process, we reconstruct the heart rate, peripheral resistance and pulse pressure, and compare the reconstructed values to the observed values. The reconstruction is based on Eqs 24–26, where we define: Hr¯(t)=Hrmin+(Hrmax-Hrmin)·Stot(t),(33) RC¯(t)=R˜min+(R˜max-R˜min)·Rmodulation(t),(34) Pp¯(t)=[K˜min+ΔK˜·S(t)]·P^v(t)P^a(t)-P^v(t),(35) where Hr¯(t), RC¯(t) and Pp¯(t) are the reconstructed values of the heart rate, vascular resistance and pulse pressure respectively. S2A–S2C Fig show the reconstruction for a randomly chosen 1% of the whole data-set which is used in Fig 9. Each dot is a single time point of one patient (all time points of a given patient are represented by the same color). Horizontal axis represents the observed value (Hr^, RC^, Pp^ in panels A, B, C respectively), and vertical axis represents the reconstructed value (Hr¯, RC¯, Pp¯ in panels A,B, C respectively). The Pearson correlation coeffici [file pcbi.1010835.s002.tiff]

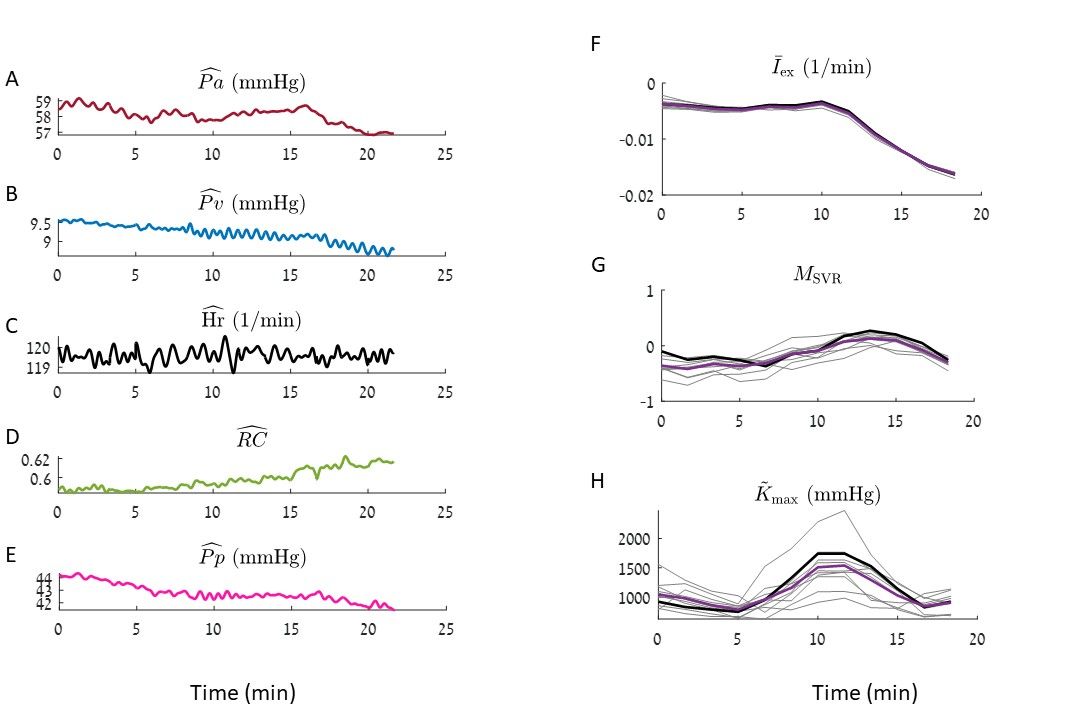

Supplement: S3 Fig — shows the results of the optimization process for different start points supplied to the optimization function. The illustration is done for patient number 1 which is identified in a hypovolemic shock state and is presented in Fig 5. Panels A-E present the extracted observables. Panels F-H present the optimal parameters obtained for realizations of the optimization process with different start points which are supplied to the optimization function. Grey lines denote for different start points, black lines are the results that appear in Fig 5. Purple lines are the average of all the realizations with different start points. It can be seen that the optimization algorithm which is used in this work to fit the iCVS model is affected by the supplied start points. However, when averaging across different initial conditions the results are concordant with the labeled shock state (hypovolemic in this case). In addition, in each single realization the average Iex is negative. We used Matlab version 2018b, running on 8 i7–8550U CPU @ 1.8GHz, 16.0GB RAM. The running time for running our optimization routine on a single interval with multiple initialization sets of parameters is 6 to 8 minutes. However, we believe that the running time can be shorter using a more optimal code, a stronger computer and maybe a different programming language. Results for patient number 1 (see Table 3) are presented. A-E.—The observables: Mean arterial pressure (A), mean venous pressure (B), heart rate (C.), RC—the peripheral resistance multiplied by the arterial compliance (D) and pulse pressure (E). F-H. Lines represent a smoothed version of the estimated parameters which are obtained in each interval (see Methods). Black line—the results which are presented in Fig 5. Purple line—average over all realizations. F. Relative intra vascular volume change (I¯ex). G. Non autonomic vascular resistance (MSVR). H. Maximal relative contractility (K˜max). Exploring identifiability: examining the abil [file pcbi.1010835.s003.tiff]

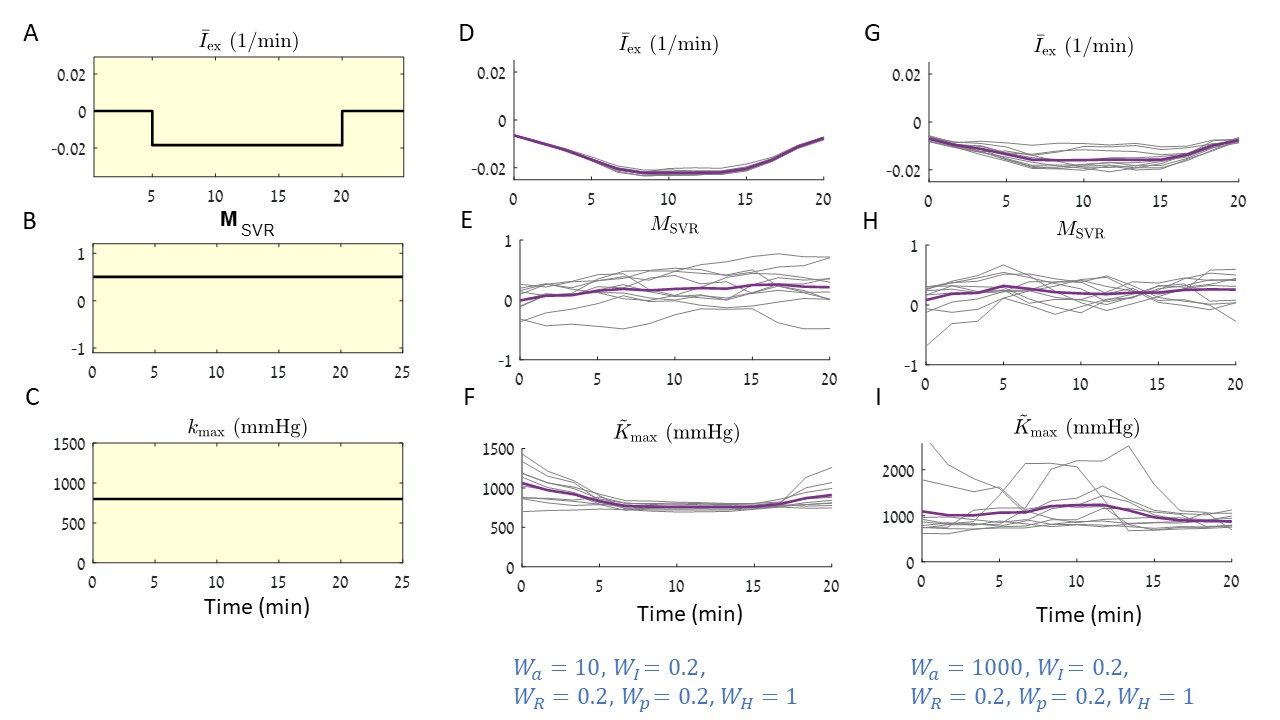

Supplement: S4 Fig — A-C.—The original parameters of the simulation chosen arbitrarily: relative intravascular volume change: I¯ex≡IexΔV0 (A), MSVR (B), and Kmax (C.). D-I. Each grey line represents a smoothed result of an estimation run obtained for each time interval (see Methods). The purple line is an average over all repeated runs (N = 5, different initialization conditions). We performed the estimation process using two different sets of weights for the optimization function to demonstrate the relative robustness of the estimation process to weight choices: Each column (D-F and G-I) represents the results that are obtained with a given set of weights as marked below. D,G Estimated relative intra vascular volume change (I¯ex). E,H Estimated non autonomic vascular resistance (MSVR). F,I estimated maximal relative contractility (K˜max). (TIFF) [file pcbi.1010835.s004.tiff]

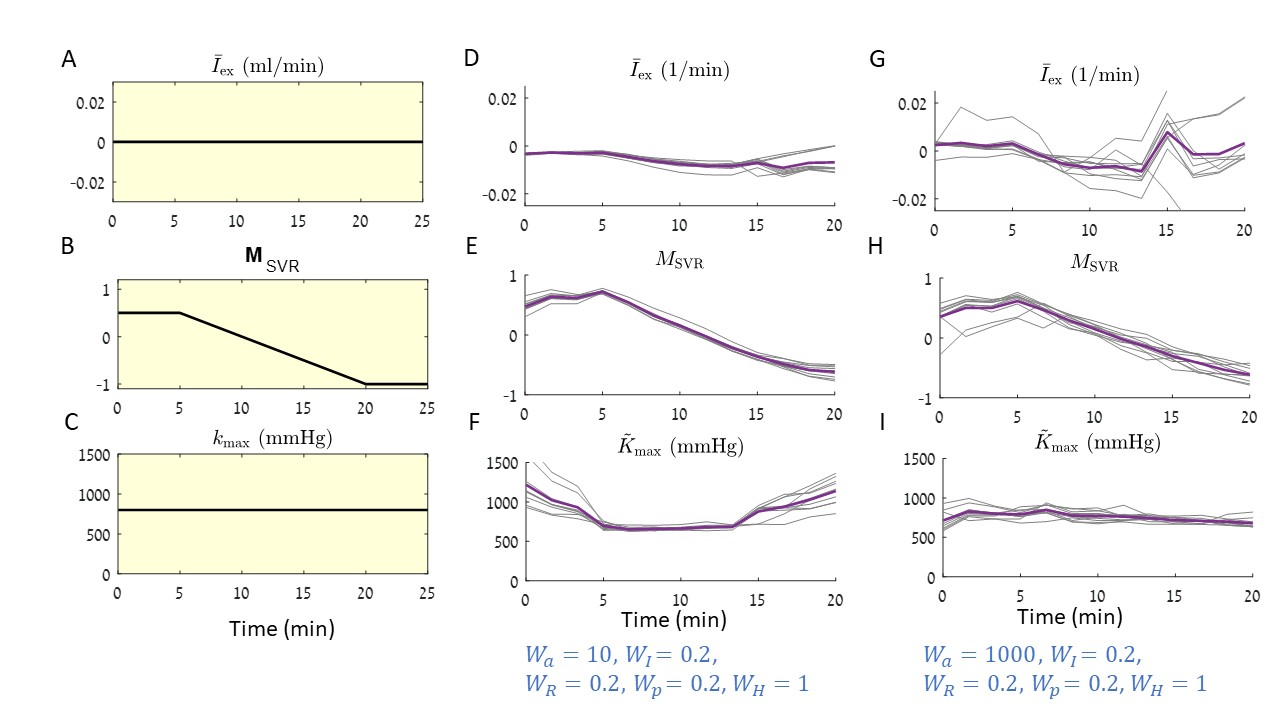

Supplement: S5 Fig — A-C.—The original parameters of the simulation chosen arbitrarily: relative intravascular volume change: I¯ex≡IexΔV0 (A), MSVR (B), and Kmax (C.). D-I. Each grey line represents a smoothed result of an estimation run obtained for each time interval (see Methods). The purple line is an average over all repeated runs (N = 5, different initialization conditions). We performed the estimation process using two different sets of weights for the optimization function to demonstrate the relative robustness of the estimation process to weight choices: Each column (D-F and G-I) represents the results that are obtained with a given set of weights as marked below. D,G Estimated relative intra vascular volume change (I¯ex). E,H Estimated non autonomic vascular resistance (MSVR). F,I estimated maximal relative contractility (K˜max). (TIFF) [file pcbi.1010835.s005.tiff]

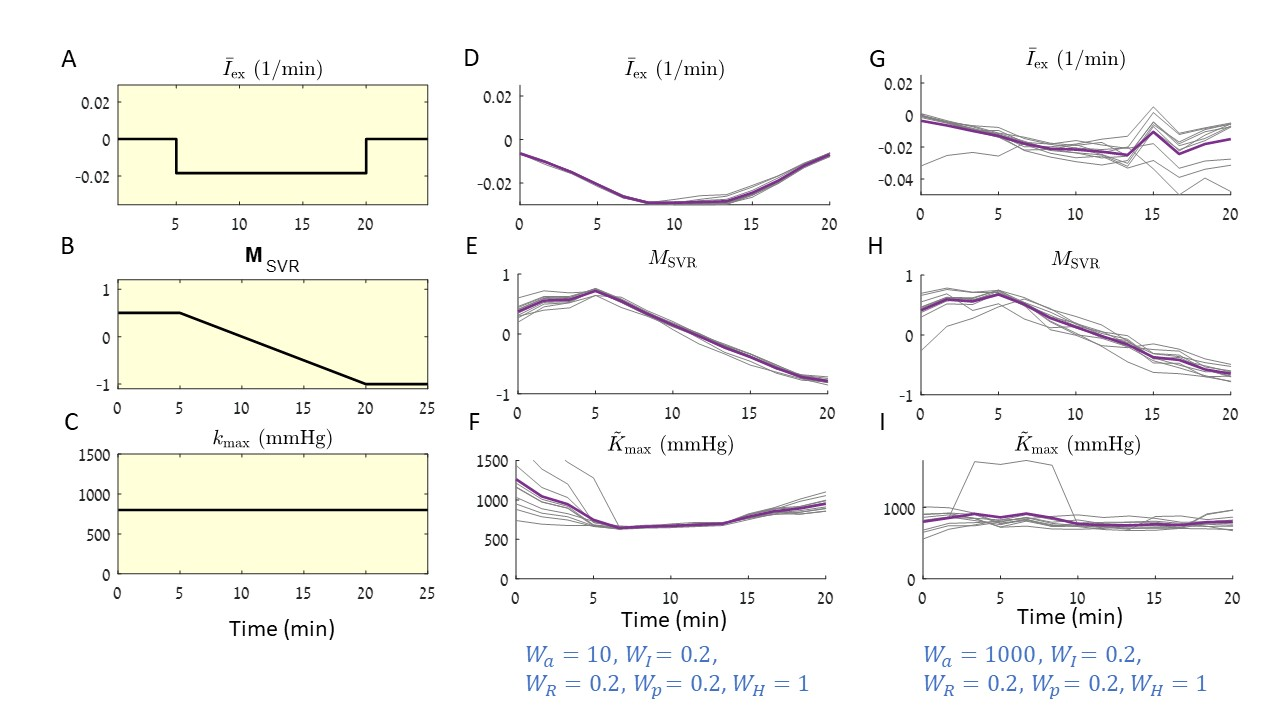

Supplement: S6 Fig — A-C.—The original parameters of the simulation chosen arbitrarily: relative intravascular volume change: I¯ex≡IexΔV0 (A), MSVR (B), and Kmax (C.). D-I. Each grey line represents a smoothed result of an estimation run obtained for each time interval (see Methods). The purple line is an average over all repeated runs (N = 5, different initialization conditions). We performed the estimation process using two different sets of weights for the optimization function to demonstrate the relative robustness of the estimation process to weight choices: Each column (D-F and G-I) represents the results that are obtained with a given set of weights as marked below. D,G Estimated relative intra vascular volume change (I¯ex). E,H Estimated non autonomic vascular resistance (MSVR). F,I estimated maximal relative contractility (K˜max). (TIFF) [file pcbi.1010835.s006.tiff]
